# Risk factors for local recurrence in patients with clinical stage II/III low rectal cancer: A multicenter retrospective cohort study in Japan

**DOI:** 10.1002/ags3.12849

**Published:** 2024-08-19

**Authors:** Takumi Kozu, Takashi Akiyoshi, Takashi Sakamoto, Tomohiro Yamaguchi, Seiichiro Yamamoto, Ryosuke Okamura, Tsuyoshi Konishi, Yoshihisa Umemoto, Koya Hida, Takeshi Naitoh, Japan Society of Laparoscopic Colorectal Surgery

**Affiliations:** ^1^ Gastroenterological Center, Department of Colorectal Surgery Cancer Institute Hospital, Japanese Foundation for Cancer Research Tokyo Japan; ^2^ Rectal Cancer Multidisciplinary Treatment Center Cancer Institute Hospital, Japanese Foundation for Cancer Research Tokyo Japan; ^3^ Department of Gastroenterological Surgery Tokai University School of Medicine Kanagawa Japan; ^4^ Department of Surgery Kyoto University Graduate School of Medicine Kyoto Japan; ^5^ Department of Colon and Rectal Surgery The University of Texas M.D. Anderson Cancer Center Houston Texas USA; ^6^ Department of Lower Gastrointestinal Surgery Kitasato University School of Medicine Kanagawa Japan

**Keywords:** lateral lymph node dissection, local recurrence, low rectal cancer, neoadjuvant treatment

## Abstract

**Background:**

Identifying risk factors for local recurrence (LR) is pivotal in optimizing rectal cancer treatment. Total mesorectal excision (TME) and lateral lymph node dissection (LLND) are the standard treatment for advanced low rectal cancer in Japan. However, large‐scale studies to evaluate risk factors for LR are limited.

**Methods:**

Data from 1479 patients with clinical stage II/III low rectal cancer below the peritoneal reflection, surgically treated between January 2010 and December 2011 across 69 hospitals, were analyzed. Fine–Gray multivariable regression modeling was used to identify risk factors associated with LR. Two models were developed: one using preoperative factors only, and the other incorporating operative and postoperative factors.

**Results:**

Across the entire cohort, the 5‐year cumulative incidence of LR was 12.3% (95% confidence interval, 10.7–14.1). The multivariable analysis associated LR with various preoperative (body mass index, distance from anal verge, cN category, and histological subtype), treatment‐related (neoadjuvant therapy, and LLND), and postoperative (pT, pN, and resection margins) risk factors. For patients without neoadjuvant treatment, LR risk was unacceptably high with two or three preoperative risk factors (body mass index ≥25 kg/m^2^, distance from anal verge ≤4.0 cm, non‐well/moderately differentiated adenocarcinoma). The 5‐year cumulative incidence of LR was 24.7% in patients treated without LLND and 22.9% in patients treated with LLND.

**Conclusion:**

This large multicenter cohort study identified some risk factors for LR in the setting where upfront TME was predominant, offering insights to optimize rectal cancer treatment.

## Graphical abstract

In this multicenter cohort study, we examined preoperative and postoperative risk factors for LR after low rectal cancer surgery. For patients without neoadjuvant treatment, LR risk was unacceptably high with two or three preoperative risk factors (BMI, distance from anal verge, histological subtype).

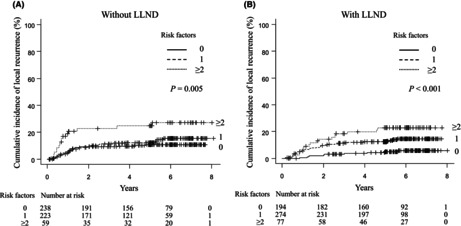

## 1 INTRODUCTION

The international standard treatment for locally advanced low rectal cancer includes preoperative neoadjuvant (chemo)radiotherapy and total mesorectal excision (TME), the combination of which reduces the incidence of local recurrence (LR) by less than 10%.^1^ Although the introduction of neoadjuvant (chemo)radiotherapy has been successful in reducing the incidence of LR, the long‐term toxicity associated with radiotherapy includes patient‐related problems such as fecal incontinence, sexual dysfunction, and secondary malignancies.^2^ A previous study found that, among patients with MRI‐defined low‐risk tumors, upfront TME resulted in as low as 3% LR,^3^ suggesting that high‐quality TME and accurate MRI staging can better select patients for whom the use of neoadjuvant (chemo)radiotherapy can be safely omitted.

Unlike in the West, Japanese guidelines for advanced lower rectal cancer recommend lateral lymph node dissection (LLND) as the standard treatment for cancers below the peritoneal reflection, with neoadjuvant (chemo)radiotherapy weakly recommended for cases with a high risk of LR.^4^ However, Japanese guidelines do not describe specifically the risk factors for considering neoadjuvant (chemo) radiotherapy.^4^ Several Japanese single‐center studies have evaluated the pre‐treatment risk factors for LR in patients with advanced low rectal cancer^5^, ^6^, ^7^, ^8^; albeit, to date, there has been no large‐scale, multi‐institutional study analyzing the risk factors for LR based on real‐world data of advanced low rectal cancer treatment in Japan.

In the current study, we aimed to clarify the preoperative and postoperative factors associated with LR in clinical stage II/III low rectal cancer below the peritoneal reflection, using a database from a large, multicenter cohort study carried out across 69 hospitals in Japan.

## 2 MATERIALS AND METHODS

### 2.1 Study population

**FIGURE 1:**
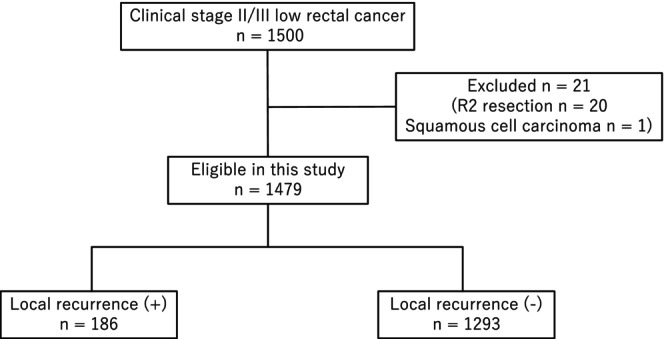
Study flowchart.

The present study involved 1500 patients with clinical stage II /III low rectal cancer with the inferior border of the tumor extending below the peritoneal reflection who underwent curative‐intent TME between January 2010 to December 2011 across 69 specialized hospitals affiliated with the Japanese Society of Laparoscopic Colorectal Surgery (UMIN registration number: 000013919). Data were collected according to the seventh edition of the classification of the Japanese Classification of Colorectal Carcinoma. The primary short‐term and mid‐term results have been reported previously.^9^ Additional retrospective surveys for histological type, long‐term prognosis, subsite of LR, and preoperative MRI findings were performed in 2017 (UMIN registration number: 000026789), and these data were also used in this study. Of the 1500 patients, we excluded 20 patients with R2 resection and one patient with squamous cell carcinoma; finally, 1479 patients were included (Figure 1). The primary and the additional survey studies were approved by the institutional review board of all participating institutions.

### 2.2 Subsite of local recurrence

LR was defined as tumor recurrence within the pelvis or perineum and was diagnosed radiologically, histologically, or clinically. LR that developed after distant recurrence was also counted. Subsites of LR were categorized as anterior, posterior, lateral (right and/or left), anastomotic, multiple subsites, others, or unknown.

### 2.3 MRI findings

Preoperative MRIs of anonymized patients were saved in DICOM format (or similar) onto CD‐R, and were captured into a research database built at Kyoto University. All collected MRI images were centrally reviewed by six experienced radiologists at Kyoto University. For patients who underwent neoadjuvant treatment, pre‐treatment MRI findings were used in this study. Of the 1479 patients, MRI data were available for 751 (51%) patients. T‐category (≤cT2, cT3, or cT4), extramural vascular invasion (mrEMVI), and circumferential resection margin (mrCRM) were evaluated. mrEMVI positivity was assessed based on criteria proposed by Brown et al.^10^ mrCRM was defined as positive when the shortest distance of the tumor to the mesorectal fascia was ≤1 mm. The number of LLNs with short‐axis size ≥5 mm was also centrally reviewed,^11^ but mesorectal lymph node involvement was not re‐evaluated. As such, for the cN category we used data collected in the initial study with diagnoses performed at each institution. A median slice thickness of 5 mm (IQR, 5–7) was used for the assessment of T2 axial images.

### 2.4 Statical analysis

Quantitative data are expressed by median values and interquartile range (IQR). Categorical variables were compared using the chi‐square test or Fisher's exact test. Comparisons of continuous variables were made using the Mann–Whitney test. Survival curves after salvage surgery are depicted using the Kaplan–Meier method, and the difference was examined using the log‐rank test. For LR, cumulative incidence was calculated using the Fine–Gray model accounting for death as a competing risk. The Fine–Gray multivariable regression model was also used to identify clinicopathological factors associated with LR. The covariates included in the models were determined from clinically potential risk factors associated with LR. We developed two models incorporating: (1) only preoperatively identifiable factors; and (2) operative and postoperative factors. Histological subtype, determined using resected specimens or biopsy results, was utilized as a factor identifiable preoperatively. A *p*‐value <0.05 was considered an indication of statistical significance. All statistical calculations were performed using JMP version 10 (SAS Institute, USA) or EZR (Saitama Medical Center, Jichi Medical University, Saitama, Japan), which is a graphical user interface for R (The R Foundation for Statistical Computing, Vienna, Austria).^12^

## 3 RESULTS

### 3.1 Patient characteristics

The baseline characteristics and pathological results for the overall cohort are shown in Table 1. Of the 1479 patients, 1019 (68.9%) were male, and the median age was 64 (IQR, 57–72) years; 746 (50.4%) patients had a tumor‐anal verge distance within 4 cm; 1365 (92.3%) patients were clinically diagnosed with T3 or T4, and 905 (61.2%) patients were clinically diagnosed with lymph node metastasis. Among the 751 patients for whom MRI data was available, mrCRM and mrEMVI were positive in 374 (49.8%) and 276 (36.8%) patients, respectively. Neoadjuvant therapy was performed in 414 (28%) patients. Of the 336 patients who received neoadjuvant (chemo)radiotherapy, 324 (96.4%) received long‐course (chemo)radiotherapy, of whom 206 (63.5%) patients received a radiation dose ≥45 Gy. Sphincter‐preserving surgery was performed in 978 (66.1%) patients, and 718 (48.5%) patients underwent LLND. The R0 resection rate was 94.9%. During the follow‐up period, 186 (12.6%) patients developed LR. The 5‐year cumulative incidence of LR was 12.3% (95% CI, 10.7–14.1). The median follow‐up period was 5.6 (IQR, 3.8–6.5) years.

**TABLE 1:** Clinicopathological characteristics of the total cohort.

Characteristics	Total (*n* = 1479)
Sex	
Male	1019 (68.9%)
Female	459 (31.0%)
Unknown	1 (0.1%)
Age (years), median (IQR)	64 (57–72)
BMI (kg/m^2^), median (IQR)	22.1 (20.0–24.5)
Distance from anal verge (cm)	
>4	733 (49.6%)
≤4	746 (50.4%)
cT category	
cT1–2	112 (7.6%)
cT3	1109 (75.0%)
cT4	256 (17.3%)
Unknown	2 (0.1%)
cN category	
cN0	570 (38.5%)
cN+/LLN‐	727 (49.2%)
cN+/LLN+	178 (12.0%)
Unknown	4 (0.3%)
Pre‐treatment CEA	
≥5 ng/mL	621 (42.0%)
<5 ng/mL	846 (57.2%)
Unknown	12 (0.8%)
mrCRM^a^	
Negative	377 (50.2%)
Positive	374 (49.8%)
mrEMVI^a^	
Negative	475 (63.2%)
Positive	276 (36.8%)
Neoadjuvant treatment	
None	1065 (72.0%)
(Chemo) radiotherapy	336 (22.7%)
Systemic chemotherapy	78 (5.3%)
Operative procedure	
Low anterior resection	700 (47.3%)
Hartmann operation	59 (4.0%)
Intersphincteric resection	219 (14.8%)
Abdominoperineal resection	468 (31.6%)
Total pelvic exenteration	33 (2.2%)
Surgical approach	
Open	909 (61.5%)
Lap	570 (38.5%)
Lateral lymph node dissection	718 (48.5%)
Postoperative complications	
≥Grade II	552 (37.3%)
Leakage/pelvic abscess	175 (11.8%)
Histological subtype	
Well/moderately differentiated adenocarcinoma	1310 (88.6%)
Others	89 (6.0%)
Unknown	80 (5.4%)
(y)pT category	
(y)pT0/Tis	20 (1.4%)
(y)pT1	54 (3.6%)
(y)pT2	274 (18.5%)
(y)pT3	982 (66.4%)
(y)pT4	146 (9.9%)
(y)pTX	3 (0.2%)
(y)pN category	
(y)pN0	781 (52.8%)
(y)pN1	378 (25.6%)
(y)pN2	184 (12.4)
(y)pN3	136 (9.2%)
Pathological lateral lymph node metastasis	125 (8.5%)
Resection margins	
R0	1403 (94.9%)
R1	76 (5.1%)
Adjuvant systemic chemotherapy	609 (41.2%)

*Note*: N3 means lateral or main lymph node metastasis.

Abbreviations: BMI, body mass index; LLN, lateral lymph node.

^a^ Cases with MRI findings *n* = 751.

### 3.2 Preoperative factors associated with LR

The results of multivariable analyses of the preoperative clinicopathological factors associated with LR for the 751 patients for whom MRI was available are shown in Table 2. BMI (≥25: HR 1.697), distance from the anal verge (≤4 cm: HR 1.745), cN category (cN+ cLLN–: HR 1.758), neoadjuvant chemotherapy (HR: 1.957), and histological subtype (other than well/moderately differentiated adenocarcinoma: HR 2.366) were independently associated with LR.

**TABLE 2:** Preoperative clinicopathological factors associated with local recurrence.

	Multivariable analysis	
	HR (95% CI)	*p*‐Value
Sex		
Male	reference	0.630
Female	1.12 (0.711–1.764)	
Age (years)	0.997 (0.978–1.016)	0.760
BMI (kg/m^2^)		
<25	reference	0.027
≥25	1.697 (1.063–2.708)	
Distance from anal verge (cm)		
>4	reference	0.019
≤4	1.745 (1.096–2.779)	
cT category (MRI)		
T1–2	reference	
T3	0.790 (0.394–1.582)	0.510
T4	1.165 (0.466–2.914)	0.740
cN category		
N0	reference	
N+/cLLN–	1.758 (1.055–2.930)	0.030
N+/cLLN+	1.295 (0.650–2.583)	0.670
Pre‐treatment CEA (ng/mL)		
<5	reference	0.600
≥5	1.124 (0.724–1.745)	
mrCRM		
Negative	reference	0.660
Positive	1.138 (0.642–2.202)	
mrEMVI		
Negative	reference	0.530
Positive	1.173 (0.710–1.938)	
Neoadjuvant therapy		
None	reference	
(Chemo) radiotherapy	0.674 (0.387–1.172)	0.160
Systemic chemotherapy	1.957 (1.002–3.822)	0.049
Histological subtype		
Well/moderately differentiated adenocarcinoma	reference	0.013
Others	2.366 (1.199–4.671)	

*Note*: Values in parentheses are 95% confidence intervals.

Abbreviations: BMI, body mass index; LLN, lateral lymph node.

### 3.3 Operative and postoperative factors associated with LR

Table 3 shows the results of the multivariable analyses, including operative and postoperative clinicopathological factors associated with LR, among the total cohort. BMI (≥25: HR 1.516), distance from anal verge (≤4 cm: HR 1.614), neoadjuvant chemotherapy (HR: 2.491), LLND (HR: 0.539), (y)pT category (T3: HR: 2.599, T4: HR: 4.107), (y)pN category (N+ LLN–: HR: 2.156, N+ LLN+: HR: 3.649), and resection margins (R1: HR:2.033) were independently associated with LR.

**TABLE 3:** Postoperative clinicopathological factors associated with local recurrence.

	Multivariable analysis	
	HR (95% CI)	*p*‐Value
Sex		
Male	reference	0.840
Female	0.967 (0.697–1.340)	
Age (years)	1.009 (0.993–1.024)	0.270
BMI (kg/m^2^)		
<25	reference	0.021
≥25	1.516 (1.065–2.158)	
Distance from anal verge (cm)		
>4	reference	0.012
≤4	1.614 (1.109–2.349)	
Pre‐treatment CEA (ng/mL)		
<5	reference	0.860
≥5	1.028 (0.753–1.403)	
Neoadjuvant therapy		
None	reference	
(Chemo) radiotherapy	1.064 (0.714–1.585)	0.760
Systemic chemotherapy	2.491 (1.449–4.283)	<0.001
Sphincter‐preserving surgery^a^		
No	reference	0.200
Yes	0.784 (0.541–1.137)	
Surgical approach		
Open	reference	0.410
Lap	0.864 (0.608–1.227)	
Lateral lymph node dissection		
No	reference	0.001
Yes	0.539 (0.371–0.783)	
Postoperative complications (leakage or pelvic abscess)		
No	reference	0.065
Yes	1.500 (0.975–2.307)	
(y)pT category		
T1–2	reference	
T3	2.599 (1.477–4.575)	<0.001
T4	4.107 (2.078–8.118)	<0.001
(y)pN category		
N0	reference	
N+/LLN–	2.156 (1.467–3.169)	<0.001
N+/LLN+	3.649 (2.051–6.493)	<0.001
Resection margins		
R0	reference	0.003
R1	2.033 (1.280–3.230)	
Histological subtype		
Well/moderately differentiated adenocarcinoma	reference	0.370
Others	1.262 (0.757–2.103)	
Adjuvant systemic chemotherapy		
No	reference	0.680
Yes	1.078 (0.755–1.540)	

*Note*: Values in parentheses are 95% confidence intervals.

Abbreviation: BMI, body mass index.

^a^ Sphincter‐preserving surgery; Low anterior resection, Hartmann operation, Intersphincteric resection.

### 3.4 Subgroup analyses in patients with upfront TME

**FIGURE 2:**
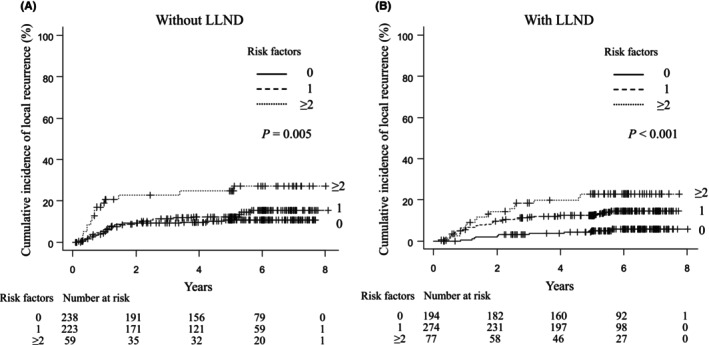
Cumulative incidence of local recurrence in patients treated with upfront total mesorectal excision without (A) or with (B) lateral lymph node dissection (LLND) according to the number of preoperative risk factors.

Of the 1065 patients who underwent upfront TME, BMI (*p* = 0.030), distance from the anal verge (*p* < 0.001), and histological subtype (*p* < 0.001) were associated with LR, but cN+/cLLN– (*p* = 0.34) was not (Figure S1). The cumulative incidence of LR was analyzed based on the presence of these three preoperative risk factors (BMI, distance from the anal verge, and histological subtype). In patients treated with upfront TME without LLND, the 5‐year cumulative incidence of LR in patients with ≥2 risk factors was 24.7%, which was significantly higher than that in patients with zero (10.7%) or one (12.3%) risk factor (Figure 2A). In patients treated with upfront TME with LLND, the 5‐year cumulative incidence of LR significantly increased with an increasing number of risk factors (0 risk factors, 4.9%; 1 risk factor, 12.4%; ≥2 risk factors, 22.9%; Figure 2B).

### 3.5 Subgroup analyses in patients with neoadjuvant (chemo) radiotherapy

The cumulative incidence of LR was also analyzed based on the presence of the aforementioned three preoperative risk factors in 336 patients who underwent neoadjuvant (chemo)radiotherapy. In patients treated with TME without LLND, the 5‐year cumulative incidence of LR was 9.7%, 13.7%, and 22.2% in patients with 0, 1, and ≥2 risk factors, respectively (Figure S2A). In patients treated with TME with LLND, the 5‐year cumulative incidence of LR was 3.6%, 7.8%, and 9.5% in patients with 0, 1, and ≥2 risk factors, respectively (Figure S2B).

### 3.6 Subsite of LR and salvage surgery

**FIGURE 3:**
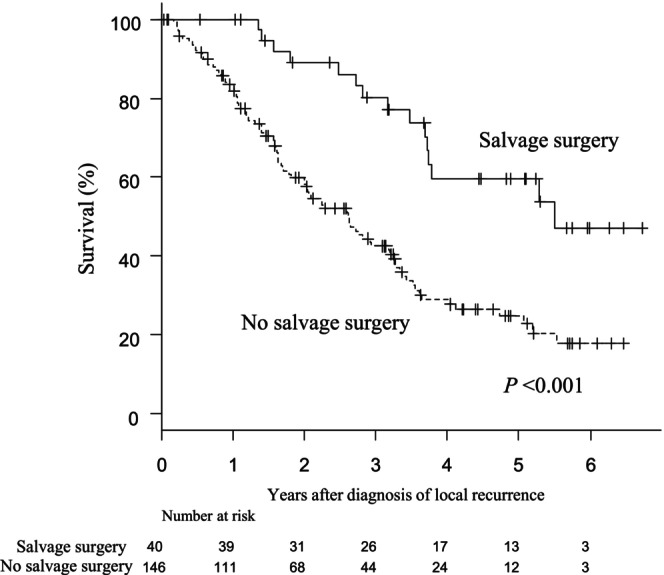
Kaplan–Meier curves for post‐recurrence survival after a diagnosis of local recurrence with or without salvage surgery for local recurrence.

Data related to the subsite of LR and the rate of salvage surgery are shown in Table 4. Lateral LR was the most common site for LR (33%), followed by presacral (23%), multiple subsites (17%), anastomotic (11%), and anterior (6%). The rate of salvage surgery was highest for anastomotic recurrence (43%), followed by anterior (36%), presacral (24%), and lateral (23%) recurrence. Of the 61 patients with lateral LR, 37 (61%) underwent LLND, while 24 (39%) did not. Table S1 shows the subsite of LR according to upfront TME with or without LLND or neoadjuvant (chemo) radiotherapy with or without LLND. Anastomotic LR was more common in patients with upfront TME without LLND compared to those with upfront TME with LLND (2.3% vs. 0.4%, *p* = 0.006). Among patients with LR, salvage surgery was significantly associated with better post‐recurrence survival compared to those without salvage surgery (5‐year post‐recurrence survival of 59.7% vs. 24.5%; *p* < 0.001) (Figure 3).

**TABLE 4:** Salvage surgery rate by local recurrence subsite.

	Local recurrence *n* (%)	Salvage surgery *n* (%)
All	186	40 (22)
Presacral	42 (23)	10 (24)
Anterior	11 (6)	4 (36)
Anastomotic	21 (11)	9 (43)
Lateral	61 (33)	14 (23)
Multiple	32 (17)	3 (9)
Others	5 (3)	0 (0)
Unknown	14 (8)	0 (0)

## 4 DISCUSSION

This study is the largest Japanese multi‐institutional study to examine the frequency of and risk factors for LR among patients with clinical stage II/III low rectal cancer below the peritoneal reflection. Upfront TME was performed in 72% of patients, with a frequency of additional LLND treatment of 48.5%; these data reflect the predominant treatment strategies for advanced lower rectal cancer at the major Japanese institutions during the study period (2010–2011).^13^ LR was observed in 12.6% of the total cohort, and we identified various preoperative (BMI, distance from anal verge, cN category, and histological subtype), treatment‐related (neoadjuvant therapy, and LLND), and postoperative (ypT and ypN category, and resection margins) risk factors associated with the development of LR.

In this study, BMI ≥25 kg/m^2^ of Japanese patients was associated with LR; we surmise that being overweight may lead to a deterioration in TME quality, thereby potentially increasing LR. Further, we showed that a lower distance from the anal verge (≤4 cm) and having a histological subtype other than well/moderately differentiated adenocarcinoma were also associated with LR. Distance from the anal verge and histological subtype have both been reported in previous studies as potential risk factors for LR,^5^, ^6^, ^8^, ^14^ whereas reports depicting associations between BMI and LR are more controversial.^15^, ^16^, ^17^ One study suggested a significant association between BMI and LR solely in the lower rectum.^15^ Some studies have reported no association between BMI and LR and even improved survival in patients with a high BMI,^17^, ^18^, ^19^, ^20^ however, these studies included stage I patients or those with middle/upper rectal cancer. Other studies have reported higher LR in clinical stage II/III rectal cancer patients with a high BMI who were treated with neoadjuvant CRT,^16^, ^21^ which is consistent with our results. These differences might be attributed to variations in patient characteristics, treatment strategies, and the surgical experience with TME for patients with a high BMI. In the present analysis, we found that, among patients undergoing upfront TME, the LR rate for those with two or more preoperative risk factors (BMI, distance from the anal verge, and histological type) was unacceptably high (≥20%), regardless of whether LLND was performed. As such, we suggest that neoadjuvant (chemo) radiotherapy should be considered for patients with ≥2 preoperative risk factors.

On the other hand, pre‐treatment MRI factors, such as cT category, mrCRM, and mrEMVI, were not associated with LR; this contrasts with previous studies showing that mrCRM and mrEMVI were important predictors of LR.^3^, ^22^, ^23^, ^24^ One possible reason for this discrepancy might be the lack of standardization among MRI protocols along with variations in the quality of MRI images across institutions. Furthermore, our study is a retrospective study using data from over a decade ago, with MRI data available for only about half of the patients. Standardized MRI protocols include high‐resolution T2‐weighted images perpendicular to the tumor with a section thickness less than 3 mm^25^; however, only 6.3% of T2‐axial images in this study had a section with slice thickness ≤3 mm. Pathological T and N categories, and positive resection margins were associated with LR in the current study, the findings of which were compatible with previous studies.^8^, ^26^, ^27^

In Western countries, several clinical trials have shown the efficacy of neoadjuvant (chemo)radiotherapy in reducing LR for locally advanced rectal cancer.^1^, ^28^ In this study, about 23% of patients underwent neoadjuvant (chemo)radiotherapy; however, neoadjuvant (chemo)radiotherapy was not significantly associated with lower rates of LR, particularly in patients with TME without LLND. This study is retrospective, and we cannot rule out that patients treated with (chemo)radiotherapy might have had a higher proportion of other non‐adjustable risk factors for LR (such as more extramural spread of the tumor). The radiation dose may also have been insufficient; about one‐third of the patients who were treated with long‐course (chemo)radiotherapy received a dose of less than 45 Gy.

We also found neoadjuvant chemotherapy to be significantly associated with more LR in this study. A recent randomized trial (PROSPECT trial) showed that neoadjuvant chemotherapy was not inferior to neoadjuvant chemoradiotherapy in terms of disease‐free survival in patients with T2N1 or T3N0–1 rectal cancer with radial margins >3 mm who were also eligible for sphincter‐sparing surgery.^29^ However, many patients included in this study did not meet the inclusion criteria of the PROSPECT trial, and neoadjuvant chemotherapy for such patients might be insufficient for reducing LR.

The inclusion of LLND as a treatment strategy was associated with a lower incidence of LR in the current study. Similar findings were found in the JCOG0212 study, which measured significantly lower LR in the lateral pelvis in patients treated with LLND than in patients treated with TME alone in the absence of enlarged LLNs.^30^ Our previous study showed that LLND offers a survival benefit for patients with short‐axis LLNs ≥5 mm.^11^ In this study, the lateral pelvis was the most common site of LR, but about two‐thirds of LR occurred in locations other than the lateral pelvis; this finding suggests that multidisciplinary treatment besides LLND is necessary to further reduce LR, particularly in patients with high‐risk factors for LR. Further studies are necessary to determine which patients would most benefit from neoadjuvant (chemo)radiotherapy or the more recently used total neoadjuvant therapy^31^, ^32^ among patients with advanced low rectal cancer below the peritoneal reflection.

This study has several limitations. First, due to the availability of MRI data for only about half of the patients, there was a much smaller sample size for the analysis of preoperative MRI factors associated with LR, which may have affected the results. Second, this is a retrospective study and is influenced by selection bias and potential unknown confounding factors. Third, the neoadjuvant treatment regimen across institutions was not uniform. Finally, although the impact of LLND on LR is considered significantly different between prophylactic and therapeutic LLND, this study did not analyze them separately.

In conclusion, our multicenter study identified some risk factors for LR in patients with advanced low rectal cancer below the peritoneal reflection. The unacceptably high LR rate in patients treated without neoadjuvant treatment and with ≥2 preoperative risk factors highlight the need for treatment optimization to reduce LR. We suggest that neoadjuvant (chemo) radiotherapy should be considered for patients with ≥2 preoperative risk factors.

## AUTHOR CONTRIBUTIONS

Drafting of article: TK and TA. Study conception and design: TK and TA. Acquisition of data: all authors. Analysis and interpretation of data: TK, TA and TS. Critical revision of article: all authors.

## FUNDING INFORMATION

This work was supported in part by Japan Society of Clinical Oncology, and the Japanese Foundation for Research and Promotion of Endoscopy.

## CONFLICT OF INTEREST STATEMENT

The authors declare no conflicts of interest for this article.

## ETHICS STATEMENT

Approval of the research protocol: The protocol for this research project has been approved by the institutional ethics committees of the participating institutions and it conforms to the provisions of the Declaration of Helsinki.

Informed Consent: N/A.

Registry and the Registration No. of the study/trial: The project was registered in the UMIN Clinical Trials Registry (UMIN000013919 and UMIN000026789).

Animal Studies: N/A.

## Supporting information


Figure S1.

Figure S2.

Table S1.
