# Supplementary figures and images for: Risk factors for local recurrence in patients with clinical stage II/III low rectal cancer: A multicenter retrospective cohort study in Japan
Source: Ann Gastroenterol Surg. 2024 Aug 19;9(1):128–36. doi: 10.1002/ags3.12849 (PMC11693533; doi:10.1002/ags3.12849)

Supplementary Figure 2

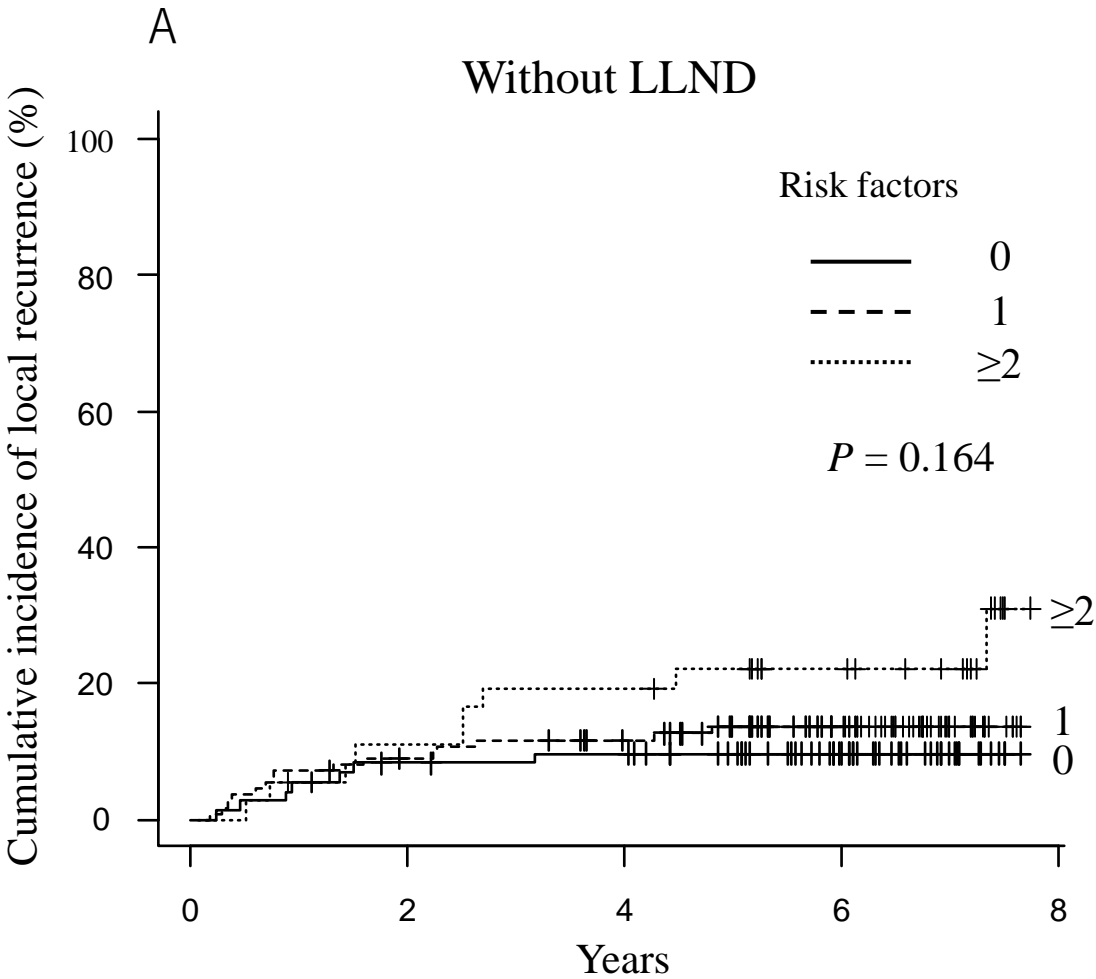

| Risk factors | Number at risk |    |    |    |   |  |
|--------------|----------------|----|----|----|---|--|
| 0            | 73             | 64 | 57 | 30 | 0 |  |
| 1            | 112            | 95 | 78 | 51 | 0 |  |
| $\geq 2$     | 37             | 31 | 27 | 18 | 0 |  |

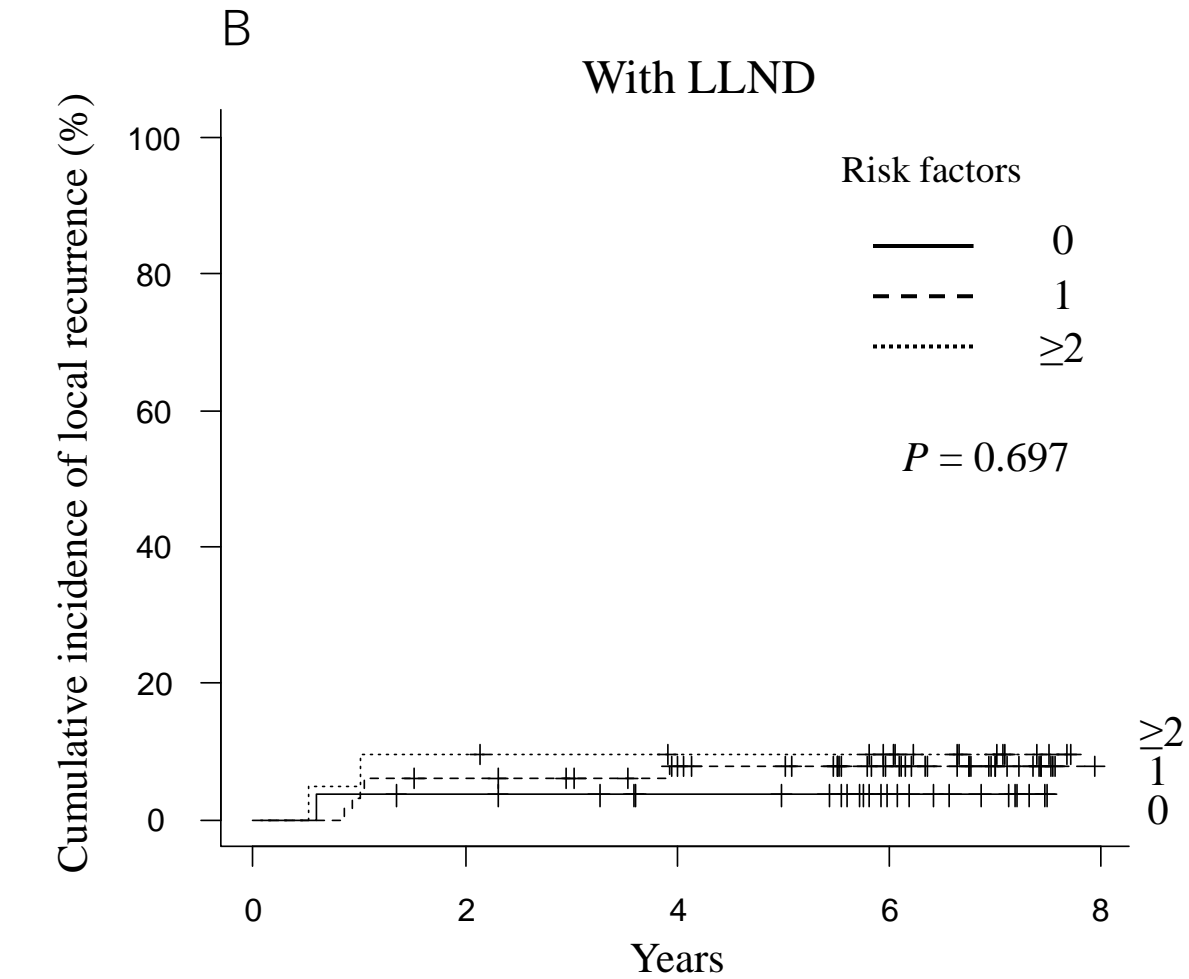

| Risk factors | Number at risk |    |    |    |   |  |
|--------------|----------------|----|----|----|---|--|
| 0            | 28             | 26 | 20 | 11 | 0 |  |
| 1            | 65             | 57 | 44 | 28 | 0 |  |
| $\geq 2$     | 21             | 18 | 14 | 12 | 0 |  |

Supplement: Supplementary file 2 — Figure S2. [file AGS3-9-128-s003.pdf]
