# Supplementary material for: Risk factors for local recurrence in patients with clinical stage II/III low rectal cancer: A multicenter retrospective cohort study in Japan
Source: Ann Gastroenterol Surg. 2024 Aug 19;9(1):128–36. doi: 10.1002/ags3.12849 (PMC11693533; doi:10.1002/ags3.12849)
Supplement: Supplementary file 3 — Table S1. [file AGS3-9-128-s002.docx]

|  | **Upfront surgery** | |  | **Neoadjuvant (chemo)radiotherapy** | |  |
| --- | --- | --- | --- | --- | --- | --- |
|  | **TME  n (%)** | **TME + LLND  n (%)** | ***P* value** | **TME n (%)** | **TME + LLND n (%)** | ***P* value** |
| All | 69 (13.3) | 64 (11.7) | 0.460 | 31 (14.0) | 8 (7) | 0.072 |
| Presacral | 17 (3.3) | 15 (2.8) | 0.720 | 8 (3.6) | 2 (1.8) | 0.504 |
| Anterior | 1 (0.2) | 4 (0.7) | 0.375 | 4 (1.8) | 0 (0) | 0.304 |
| Anastomotic | 12 (2.3) | 2 (0.4) | 0.006 | 5 (2.3) | 1 (0.9) | 0.668 |
| Lateral | 15 (2.9) | 27 (5.0) | 0.086 | 9 (4.1) | 3 (2.6) | 0.757 |
| Multiple | 14 (2.7) | 11 (2.0) | 0.546 | 4 (1.8) | 0 (0) | 0.304 |
| Others | 3 (0.6) | 0 (0) | 0.116 | 0 (0) | 1 (0.9) | 0.339 |
| Unknown | 7 (1.3) | 5 (0.9) | 0.571 | 1 (0.5) | 1 (0.9) | 1.000 |

Supplementary Table 1. Local recurrence subsite according to upfront TME with or without LLND or neoadjuvant (chemo)radiotherapy with or without LLND

TME, total mesorectal excision; LLND, lateral lymph node dissection.
